# Current concepts in atherosclerosis

**DOI:** 10.1007/s12055-018-0699-y

**Published:** 2018-10-20

**Authors:** Mohammad Alkhalil, Robin P. Choudhury

**Affiliations:** 1 Acute Vascular Imaging Centre, Division of Cardiovascular Medicine, Radcliffe Department of Medicine, University of Oxford, John Radcliffe Hospital, Oxford, OX3 9DU UK; 2 Cardiology Department, Royal Victoria Hospital, Belfast, UK

**Keywords:** Atherosclerosis, Lipids, Inflammation

## Abstract

Atherosclerosis is a complex disease process. It is increasingly recognised that both lipoprotein retention and inflammatory cellular components are intricately related in the initiation and development of atherosclerotic plaque. LDL-c (cholesterol) has been long established as a cause for atherosclerosis; additionally, inflammatory cells such as monocytes and subsequently foam cells have also been directly linked to the progression of atherosclerotic disease. Emerging data suggest that structures outside vascular intima and media are also closely related to atherosclerosis. Perivascular adipose tissue (PVAT) may be a determinant of the inflammatory status of the atherosclerotic plaque. All these features are becoming extremely relevant as therapies against atherosclerosis are targeting both lipid retention and inflammation. Recently, there has been some success in these novel therapies, such as the proprotein convertase subtilisin-kexin type 9 (PCSK-9) inhibitor evolocumab and the interleukin-1ß neutralising antibody, canakinumab, in reducing cardiovascular events when added to standard therapy such as statin. This review will discuss the pathogenesis of atherosclerosis, including some novel features, and its management using new anti-atherosclerotic drugs.

## Introduction

Atherosclerosis is a complex disease with multiple processes implicated in early atherogenesis. Lipoprotein retention within the intima of the vascular wall coupled with inflammatory flux of monocytes and lymphocytes in addition to changes in endothelial function and migration of vascular smooth muscle cells leads to the formation of atherosclerotic plaque [1]. Early events in initiating atherosclerosis are the deposition and retention of apoB-containing lipoproteins, and endothelial cell dysfunction, especially at branch regions of large and medium-sized arteries [2]. Using state-of-art imaging techniques, in vivo human studies demonstrated that arterial walls, at the bifurcation, contained larger lipid deposition than non-bifurcating regions [3]. This susceptibility has been attributed to the loss of laminar flow and changes in shear stress on endothelium with subsequent intra and inter-cellular alterations in endothelial functions [4]. These changes trigger inflammatory response and are coupled with increased rate of lipoprotein accumulation and extracellular matrix deposition leading to intimal thickening and facilitating the process of atherosclerotic plaque formation [5]. To the contrary, intra-myocardial coronary segments or myocardial bridging have consistently been atherosclerosis-free [6]. The insulation of these segments from perivascular adipose tissue (PVAT) has instigated a new paradigm where the process of developing atherosclerotic plaque is initiated at the outside of the vessel wall and progressed towards the intima, an ‘outside-in’ theory [6]. Regardless of whether it is implicated in the initiation of atherosclerosis, PVAT is increasingly recognised as an active endocrine organ that is closely linked to the development of atherosclerosis [7, 8].

Progression of atherosclerotic plaque is accelerated by the increased number of intimal monocytes and the ingestion of LDL particles by macrophages generating lipid-rich foam cells [9]. The endurance of lipid droplets secondary to the failure to clear dying foam cells and macrophages triggers further inflammation and subsequently larger accumulation of foam cells layers and extracellular lipid. This formation is the first macroscopic appearance of atherosclerosis and is referred to as fatty streak lesions [9]. These intimal changes not only start early in adolescents but also have been shown to be predictive of occlusive vascular events [10]. The clinical manifestations of these occlusive events, such as acute coronary syndromes (ACS) and cerebrovascular events (CVA), are commonly a consequence of an abrupt plaque rupture which can sometimes precipitate occlusive luminal thrombus [11]. Despite the progress in understanding the pathology of atherosclerosis and the advancement in mechanical and pharmacological therapies, atherosclerosis-related diseases continue to be leading cause of mortality worldwide [12]. In this review, we will discuss the pathogenesis of atherosclerosis including new and novel mechanisms related to the development and clinical manifestations of atherosclerotic plaque, in addition to the recent pharmacological advances in tackling this disease.

## Role of lipid accumulation in the pathogenesis of atherosclerosis

Lipoprotein retention is the cornerstone of the development of atherosclerosis [1, 11]. The significance of role of cholesterol was established very early by Anitschkow and Chatalow [13]. They demonstrated that high cholesterol diet was associated with the formation of atherosclerotic lesions within the arterial intima of rabbits [13]. Regardless of the stage of atherosclerosis development, Anitschkow and Chatalow demonstrated that progression of atherosclerotic plaques was proportional to the degree of cholesterol uptake [13]. Subsequent imaging studies of arterial intima confirmed interweaved collagen fibres with trapped LDL particles structure in cholesterol-fed rabbits [14]. Succeeding histological studies established the association between large plaque cholesterol burden and cardiovascular events [15]. In fact, plaques with a large lipid core were four times more likely to be associated with ruptured plaques (OR 4.03, 95% confidence interval 2.35 to 6.09, *P* < 0.001) [15]. Moreover, plaque lipid was considered to be one of the best discriminators to determine the vulnerability of atherosclerotic plaques [15, 16]. Likewise, large epidemiological studies established a strong relationship between serum cholesterol level and vascular events [17]. The Framingham Heart Study showed that LDL-c is directly related to the risk of cardiovascular disease (CVD) [17]. In addition, evidence from Mendelian randomisation revealed that alleles associated with LDL-c were associated with an increased risk of CVD [18]. Subsequently, pharmacological therapies targeting LDL-c, via statin and recently non-statin drugs, demonstrated significant reduction in future vascular events [19–21]. However, it is important to recognise the lack of relationship between plasma cholesterol level, a strong epidemiological risk factor, and plaque cholesterol burden, a marker of high-risk atherosclerotic plaque [22]. While plasma cholesterol level has been a predominant feature in atherosclerosis, other processes were suggested as prerequisites to the initiation of atherosclerotic plaque. The response-to-injury hypothesis was proposed to support the role of endothelium in instigating atherosclerosis. It was originally hypothesised that arterial endothelial cells, through their highly inter-digitated junctions, form a barrier that preclude the passage of atherogenetic molecules, and therefore, endothelial denudation or injury would promote atherosclerosis [23, 24]. Nonetheless, subsequent studies have demonstrated that the endothelium covering atherosclerotic lesions at early stages of development is, in fact, structurally intact [1, 25]. The response-to-injury hypothesis was consequently modified, and alterations in endothelial function were proposed as essential stimulus for atherosclerosis [26]. The early implication of endothelial dysfunction in atherosclerosis prior to any structural changes detected on invasive or non-invasive imaging modalities supports endothelium’s key role in early atherogenesis [26]. Following cholesterol-rich diet, there was a strongly positive correlation between the permeability to LDL-c in segments of rabbit’s aorta and the cholesterol deposition within the same aortic segment [27]. The increased permeability of atherogenic lipoprotein was suggested as a marker of endothelial dysfunction and an evidence of early involvement in triggering atherosclerosis [1, 27]. However, two observations challenged the refined response-to-injury hypothesis and the proposed mechanism of increased cholesterol permeability into the vascular intima. Firstly, in vivo studies demonstrated that LDL entry rate is not different between atherosclerosis susceptible and resistant sites [28]. In some reports, there was even a decrease in LDL entry rate into atherosclerosis susceptible sites, highlighting the lack of causality between endothelial permeability and the initiation of atherosclerosis [28]. Secondly, the flux of LDL-c into the arterial wall, even in normal vascular wall, is significantly larger than the accumulation of cholesterol within the intima. This suggests a secondary process affecting the rate of lipid deposition and probably related to cholesterol efflux from the vascular wall [1, 29]. Reverse cholesterol transport was early recognised, and the reverse correlation between HDL-c level and vascular events was suggestive of HDL-mediated process in cholesterol efflux from vascular wall [30]. Yet, recent evidence have suggested the lack of a simple causal role of HDL-c in protection from atherosclerosis. Mendelian randomisation studies have demonstrated that alleles linked with HDL-c are not associated with future cardiovascular events after multiple adjustments [18]. Moreover, a genetic variant of scavenger receptor B1 was showed to cause profound increase in HDL-c but it did not decrease the risk of CVD [31]. To the contrary, carriers of P376L variant had significantly increased risk of future events (odds ratio (OR) 1.79, *P* = 0.018) [31]. Subsequently, pharmacological therapies to raise HDL-c were unsuccessful in reducing cardiovascular events [32]. This questions the hypothesis that measurement of HDL-c is a useful metric of the functional effects of HDL particles. In fact, more recent data quantifying HDL function by measuring cholesterol efflux from macrophages have demonstrated inverse association with atherosclerosis burden and more importantly with vascular events, independent of HDL-c plasma level [33].

## The inflammatory cellular component of atherosclerosis

LDL-c retention within the intima of vascular wall triggers a cascade of inflammatory cellular influx mainly monocytes. The susceptible LDL particles to oxidation are chemo-attractant to monocytes, lymphocytes and vascular smooth muscle cells [34]. In addition, there are changes in endothelial function by increasing the expression of cell adhesions molecules, such as vascular cell adhesion molecule-1 (VCAM-1) to recruit more circulating monocytes from the bone marrow or the spleen [34]. Following transmigration via chemokine receptor families (CCR2, CCR5 and CX3C), monocytes can differentiate and locally proliferate into macrophages and also directly influence the phenotype of in-situ macrophages [34]. The recruited monocytes have been largely classified into two major subsets CD14^+^CD16^−^ and CD14^low^CD16^+^. The former one is presumably more inflammatory and classically considered to be the source of M1 macrophages responsible for marinating chronic inflammatory state [35]. This crude classification is based on molecules on cellular membrane and measures using genomic and metabolomic analyses may be better reflection of cellular function or even origin. This is important as cholesterol-loaded vascular smooth muscle cells not only express macrophage markers but lose their differentiation markers [36]. The functional properties of these macrophage-appearing smooth muscle cells were not identical to classical macrophages [37]. Vascular smooth muscle cells have been implicated in enhancing the expression of LDL particle-retaining molecules [1]. The proliferation and migration of vascular smooth muscle cells from the medial layer into the intima is influenced by release of growth factors from recruited leucocytes and activated endothelial cells. These cells are either transformed into macrophage-like cells or contribute to the formation of extracellular proteoglycan [38]. Extracellular proteoglycan has an important role in retaining LDL particles and, thus, maintaining the trigger for inflammatory response [1]. In addition, proteoglycan confers the structural integrity of the atherosclerotic plaque, including the overlying collagen-rich fibrous cap. The accumulating LDL-c is initially cleared up by macrophages, transforming them into foam cells. When the accumulation of lipid exceeds the clearance rate, a necrotic core within the atherosclerotic plaque can become apparent [34].

Despite the central role of monocytes/ macrophages, other cell types take part in the development of atherosclerosis, including dendritic, eosinophils, mast cells and lymphocytes. The adaptive immune system (including T and B lymphocytes) secretes a number of cytokines, depending on their local environment, which has an impact on atherosclerotic plaque. These cytokines are either pro-inflammatory like IL1ß, IL6 and TNF or promote resolution of inflammation such as IL4 and IL10 [34]. The pro-inflammatory cytokines or T_h_1 response also enhances protease and chemokine secretion, with less formation of collagen fibre, upregulation of adhesion molecules and activation of macrophages and endothelial cells [39]. On the other hand, anti-inflammatory cytokines, particularly IL4, or T_h_2 response has been less clear with some studies suggesting protective role while others showing neutral effects [39].

Circulating microparticles have also been shown to play a significant role in the pathogenesis of atherosclerosis [34]. These vesicular bodies derived from platelets, monocytes and red blood cells are important in cell-to-cell signalling especially in context of inflammation [40]. More importantly, characterising these particles according to their content of protein, mRNA and miRNA, has also been significant in the function of cellular adhesions molecules, such as VCAM, and activation of macrophages within atherosclerotic plaque [34, 41]. Overall, this underlines the complexity of atherosclerosis disease and highlights that it is not an exclusive disease of lipid retention. In fact, there are new biomarkers that recently have been implicated in the development of atherosclerotic plaque.

## Novel mechanisms in the progression of atherosclerosis and its clinical manifestations

Atherosclerosis is a disease affecting the vascular media and intima. Nonetheless, structures like the adventitia, through vasa vasorum, or more recently PVAT have been recognised as active participants in the progression of atherosclerotic plaque [8]. The primary function of microvasculature within the adventitial layers is to provide nutrients to the cells of the arterial wall [42]. However, during plaque development, oxidised phospholipids can stimulate abnormal formation of vasa vasorum leading to neovascularization through the production of vascular endothelial growth factor (VEGF) [42]. These immature microvessels are prone to leak causing intra-plaque haemorrhage which is a recognised feature of plaque vulnerability. Moreover, these neo-vessels can enhance monocyte accumulation and cytokine release affecting the vascular intima and further aggravating inflammation within the atherosclerotic plaque. Some of these cytokines are secreted from adipose tissue surrounding vascular structure, and this direct relationship with the adventitia has suggested a significant role of PVAT in the development of atherosclerosis [7, 42]. PVAT, through the release of monocyte chemo-attractant protein-1 (MCP-1), promotes differentiation of monocytes into pro-inflammatory macrophages [43]. This may contribute to the increased levels of pro-inflammatory cytokines in patients with coronary artery disease [6]. Further studies have demonstrated that pro-inflammatory proteins secreted from PVAT can stimulate inflammation in the vascular wall perpetuating atherosclerosis process [6]. Using different imaging modalities, the whole volume of PVAT has been associated with the severity of atherosclerosis burden [6]. This has been attributed to the close connectivity between PVAT and vascular wall. However, using dedicated cardiovascular magnetic resonance imaging (CMR) to assess the circumferential relationship, PVAT was weakly correlated with atherosclerosis burden measured as vessel wall thickness [7]. This conclusion challenged the proposal of paracrine route of PVAT connection with atherosclerosis. Nevertheless, this disassociation did not rule out other potential mechanisms such as the role of PVAT characteristics, rather than its total volume, in atherosclerosis. This hypothesis was tested using computed tomography (CT) and relied on measuring Hounsfield unit of PVAT to reflect its inflammatory status [8]. Changes in adipocytes in responses to inflammation in terms of cellular size and lipid content are potentially detectable on CT using a novel method to measure PVAT composition (fat attenuation index) [8]. This index has been proposed as an imaging biomarker to reflect inflammatory status within the vessel wall [8]. Overall, the role of PVAT in atherosclerosis is increasingly considered in the progression of atherosclerosis.

## Management of atherosclerosis—standard and novel therapies

Despite better understanding into the pathogenesis of atherosclerosis, it continues to be the most common cause of death worldwide [22]. Targeting lipoprotein and inflammatory pathways has been the main approaches in tackling atherosclerosis. 3-Hydroxy-methylglutaryl coenzyme A (HMG Co-A) reductase inhibitors, statins, have been the standard therapeutic option in atherosclerosis-related diseases [44]. The benefits of statins have been attributed to reduction in LDL-c. The magnitude of benefits, using statins, was proportional to the level of attained LDL-c, and these benefits were noted in both primary and secondary prevention settings [45–47]. The first statin trial to demonstrate survival benefits was the Scandinavian Simvastatin Survival Study (4S) [45]. This landmark study recruited 4444 patients with coronary artery disease (CAD) and demonstrated 3.7% absolute mortality reduction after 5.4 years of follow-up [45]. The Pravastatin or Atorvastatin Evaluation and Infection Therapy Thrombolysis in Myocardial Infarction 22 (PROVE IT-TIMI 22) and Treating to New Targets (TNT) studies [46, 48] demonstrated incremental benefits from reducing LDL-c using intensive statin treatments. This relationship between statin-induced LDL-c reduction and vascular events was borne out in a large meta-analysis, including 170,000 patients from 26 randomised trials. In this meta-analysis conducted by the ‘Cholesterol Treatment Trialists’ (CTT) collaboration, statins were shown to reduce the relative risk of major vascular events by 23% for every 1.0 mmol/L reduction in LDL-c [19]. The Justification for the Use of Statins in Primary Prevention: an Intervention Trial Evaluating Rosuvastatin (JUPITER) trial demonstrated a superior reduction in cardiovascular events with further reduction in high sensitivity C-reactive protein (hsCRP) [49]. There was 79% reduction (HR 0.21, 95% CI 0.09–0.52), 65% reduction (HR 0.35, 95% CI 0.23–0.54) and 33% reduction (HR 0.67, 95% CI 0.52–0.87) in vascular events in rosuvastatin-treated individuals who achieved both LDL-c < 1.8 mmol/L and hsCRP < 1 mg/dL, LDL < 1.8 mmol/L and hsCRP < 2 mg/L and those who achieved one or neither target (LDL < 1.8 mmol/L or hsCRP < 2 mg/L), respectively [49]. The ‘pleiotropic’ effects of statins have fuelled the argument that clinical benefits of statins were derived from their impact on inflammation, in addition, to other properties on endothelial function and anti-oxidation [50].

This ongoing debate has probably put into a halt following data from ezetimibe in the Improved Reduction of Outcomes: Vytorin Efficacy International Trial (IMPROVE-IT), where incremental cardiovascular benefits were demonstrated when adding a non-statin drug [20]. Combining ezetimibe and simvastatin resulted in an absolute reduction of 2% in major cardiovascular events [20]. This reduction was attributed to attained LDL-c in the combination group of 1.4 mmol/L compared to 1.8 mmol/L in the simvastatin group after a median follow-up of 6 years. The significance of this study was twofold. Firstly, ezetimibe was the first non-statin treatment to show additional cardiovascular benefits when added to statin. Secondly, the magnitude of cardiovascular benefits in response to LDL-c was similar to previous statin studies, reinforcing the LDL hypothesis. The LDL hypothesis, as opposed to the pleiotropic hypothesis, reasons that reduction in vascular events when using statins is related to LDL-c reduction and not to statin pleiotropic effects, e.g. on inflammation or endothelial function [51]. The mechanism of ezetimibe by blocking LDL-c absorption from the intestines provided an excellent opportunity to study the impact of lowering LDL-c in isolation from statin mode of action.

More recently, PCSK9 inhibitors have led to unprecedented decrease in LDL-c which was translated into reduction in cardiovascular events [21]. PCSK9 is a glycoprotein that is secreted from the liver and plays an important role in regulating LDL-c [22, 52]. The catalytic subunit of PCSK9 binds to LDL receptors promoting its degradation and preventing it from recirculating back to the cellular membrane of hepatocytes [22, 52]. Blocking PCSK9 using monoclonal antibodies would preserve LDL receptors permitting further reduction in LDL-c [22, 52]. The FOURIER study (Further Cardiovascular Outcomes Research with PCSK9 Inhibition in Subjects with Elevated Risk) was the first trial to assess the efficacy of inhibiting PCSK9 on clinical outcomes [21]. More than 27,000 patients with high-risk atherosclerotic disease were randomised to either subcutaneous evolocumab or placebo and were followed up over median of 26 months [21]. The absolute reduction in LDL-c of almost 40 mg/dL was translated into an absolute risk reduction in cardiovascular events of 1.5%. Further prespecified analysis from the FOURIER trial demonstrated a monotonic relationship between attained LDL-c and reduction in cardiovascular events, extending to LDL-c < 20 mg/dL [53]. Most recently, data from the ODSSEY OUTCOMES study were recently presented in the American College of Cardiology Conference investigating the efficacy of alirocumab on clinical outcomes [54]. Alirocumab is another PCSK9 inhibitor which was tested in more than 18,000 patients presenting with acute coronary syndrome with baseline LDL-c of 87 mg/dL. After 2.8 years of follow-up, alirocumab decreased LDL-c to only 53 mg/dL but with similar magnitude to evolocumab in reducing cardiovascular outcomes (11.1 to 9.5%, HR 0.85, 95% CI 0.78 to 0.93, *P* < 0.001). These benefits were derived from reduction in non-fatal myocardial infarction, ischaemic stroke and unstable angina but not on cardiovascular mortality. Interestingly, there was a statistically significant reduction in all-cause mortality.

The recent advances in pharmacotherapies tackling lipoproteins were also matched with similar progress in the inflammatory pathway of atherosclerosis. Canakinumab is a human monoclonal antibody against IL1ßwhich was studied in the Canakinumab Anti-Inflammatory Thrombosis Outcomes Study (CANTOS) trial [55]. There was a significant reduction in cardiovascular events using canakinumab when tested against placebo in patients with recent acute coronary syndrome and persistent hsCRP ≥ 2 mg/dL (HR 0.85, 95% CI 0.74 to 0.98, *P* = 0.021) [55]. However, this was associated with significant increase in the rate of fatal infection (0.31 vs. 0.18 events per 100 person-years, *P* = 0.02) [55]. This landmark study illustrated that targeting inflammation is also a promising approach in reducing atherosclerosis-related risks. The effects of canakinumab on the ‘central’ inflammatory pathway of IL1, IL6, tumour necrosis factor (TNF-α) and CRP may explain the lack of success with previous anti-inflammatory drugs [56, 57]. This pathway is considered a critical mechanism in the progress and development of atherosclerosis [56, 57].

## Need for risk stratification and role of vascular imaging

The recent relative success of novel therapies in reducing cardiovascular events has promoted an important debate as to whether these drugs are applicable for the real world. These drugs have typically produced an absolute risk reduction of 1–2% of combined clinical outcomes, including death, myocardial infarction, stroke, unstable angina and coronary revascularisation. This reflects a small margin of benefits challenging their unselective use in atherosclerotic patients. Numerous factors may explain the modest effects of these drugs. Firstly, all these therapies have been tested against placebo in statin-treated patients. The significant effects when using statin in reducing cardiovascular events would make detection of any potential benefits more challenging. Moreover, the current guidelines mandate the use of anti-platelet therapy in addition to angiotensin converting enzyme inhibitors and beta blocker aggregating further reduction in vascular events [44]. This would render demonstration of possible benefits using a new drug more taxing. Finally, there is a significant heterogeneity in patients presenting with atherosclerosis-related diseases. Beyond the category of the atherosclerotic patients, i.e. presenting with stroke or myocardial infarction, it is now increasingly recognised that certain individuals have susceptibility to calcific disease, inflammation, risk of thrombosis or tendency for lipid accumulation [3, 22]. While a certain drug may be effective in tackling a specific disease substrate, the overall outcome may become ‘diluted’ and undermine the efficacy of the drug. Therefore, it becomes less surprising why these novel anti-atherosclerotic drugs have failed to show larger benefits when compared to standard therapies like statins [22, 58]. More importantly, it is now more pressing to identify a group of patients who may benefit maximally from these drugs. This is important given that these drugs produced a large number-needed-to-treat (NNT) when they were used unselectively on all-comers (1:50 for ezetimibe, 1:67 in evolocumab and 1:51 for canakinumab, in the trials cited, above). This unmanageable NNT would question the clinical suitability and cost-effectiveness of these novel therapies. Moreover, these drugs are not without any side-effects (fatal infection in canakinumab and neurocognitive impairment in PCSK9 inhibitors) [55, 59]. Therefore, there is urgent need to identify a group of patients who are at high risk to rationale the use of these novel therapies.

**Fig. 1:**
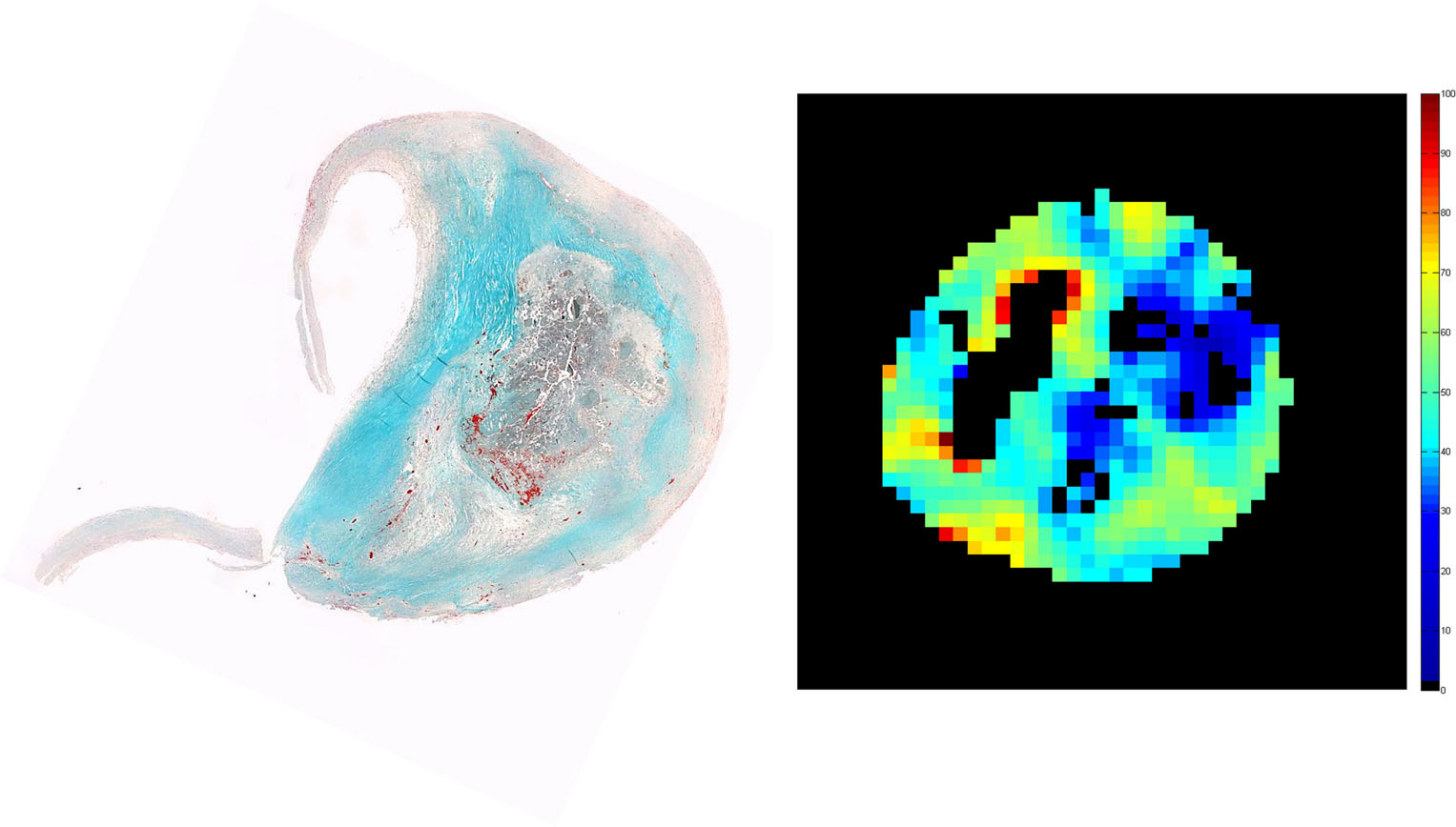
Comparison of T2 mapping and histology. T2 mapping identifies different components of atherosclerotic plaque (lipid in blue, recent intra-plaque haemorrhage in red, fibrous in green and calcium in black). Lumen is also shown in black. This is confirmed on histology (only Masson's staining is shown)

One promising strategy is to characterise atherosclerotic disease and to identify the prominent disease substrate, e.g. lipid accumulation and inflammation, and to match a drug with a mechanism of action that targets the particular substrate [3, 22]. Such a method would offer a more precise intervention, with potentially greater efficacy and more cost-effectiveness [22]. There are currently available techniques that could potentially ‘map’ individuals at risk and categorise them according to their disease characteristics (Fig. 1) [3, 7]. This strategy is yet to be tested in the future to assess whether it would be effective, but undoubtedly, it would decrease the sample size to prove efficacy, reduce costs and follow-up duration but more importantly would spare low-risk individuals from unnecessary risks.

## Conclusion

There is currently better understanding regarding the pathogenesis of atherosclerosis. Although significant progress has been made since Anitschkow’s work, the main pathways of atherosclerotic plaque continue to be lipid retention and inflammation. Novel successful therapies targeting atherosclerosis are promising, but further refinements are required prior to their application in the real world.

## References

[1] Williams KJ, Tabas I (1995). The response-to-retention hypothesis of early atherogenesis. Arterioscler Thromb Vasc Biol.

[2] Thubrikar MJ, Keller AC, Holloway PW, Nolan SP (1992). Distribution of low density lipoprotein in the branch and non-branch regions of the aorta. Atherosclerosis.

[3] Alkhalil M, Biasiolli L, Chai JT (2017). Quantification of carotid plaque lipid content with magnetic resonance T2 mapping in patients undergoing carotid endarterectomy. PLoS One.

[4] Eshtehardi P, McDaniel MC, Suo J (2012). Association of coronary wall shear stress with atherosclerotic plaque burden, composition, and distribution in patients with coronary artery disease. J Am Heart Assoc.

[5] Wang L, Luo JY, Li B, et al. Integrin-YAP/TAZ-JNK cascade mediates atheroprotective effect of unidirectional shear flow. Nature. 2016; 10.1038/nature20602. 10.1038/nature20602 27926730

[6] Verhagen SN, Visseren FL (2011). Perivascular adipose tissue as a cause of atherosclerosis. Atherosclerosis.

[7] Alkhalil M, Edmond E, Edgar L, et al. The relationship of perivascular adipose tissue and atherosclerosis in the aorta and carotid arteries, determined by magnetic resonance imaging. Diab Vasc Dis Res. 2018; 10.1177/1479164118757923. 10.1177/1479164118757923 PMC6039860 29446645

[8] Antonopoulos AS, Sanna F, Sabharwal N, et al. Detecting human coronary inflammation by imaging perivascular fat. Sci Transl Med. 2017;9. 10.1126/scitranslmed.aal2658. 10.1126/scitranslmed.aal2658 28701474

[9] Stary HC, Chandler AB, Dinsmore RE (1995). A definition of advanced types of atherosclerotic lesions and a histological classification of atherosclerosis. A report from the Committee on Vascular Lesions of the Council on Arteriosclerosis, American Heart Association. Circulation.

[10] Strong JP, Malcom GT, McMahan CA (1999). Prevalence and extent of atherosclerosis in adolescents and young adults: implications for prevention from the Pathobiological Determinants of Atherosclerosis in Youth Study. JAMA.

[11] Fuster V, Stein B, Ambrose JA, Badimon L, Badimon JJ, Chesebro JH (1990). Atherosclerotic plaque rupture and thrombosis. Evolving concepts. Circulation.

[12] GBD 2013 Mortality and Causes of Death Collaborators (2015). Global, regional, and national age-sex specific all-cause and cause-specific mortality for 240 causes of death, 1990-2013: a systematic analysis for the Global Burden of Disease Study 2013. Lancet.

[13] Finking G, Hanke H (1997). Nikolaj Nikolajewitsch Anitschkow (1885-1964) established the cholesterol-fed rabbit as a model for atherosclerosis research. Atherosclerosis.

[14] Frank JS, Fogelman AM (1989). Ultrastructure of the intima in WHHL and cholesterol-fed rabbit aortas prepared by ultra-rapid freezing and freeze-etching. J Lipid Res.

[15] Redgrave JN, Gallagher P, Lovett JK, Rothwell PM (2008). Critical cap thickness and rupture in symptomatic carotid plaques: the oxford plaque study. Stroke.

[16] Narula J, Nakano M, Virmani R (2013). Histopathologic characteristics of atherosclerotic coronary disease and implications of the findings for the invasive and noninvasive detection of vulnerable plaques. J Am Coll Cardiol.

[17] Kannel WB, Castelli WP, Gordon T (1979). Cholesterol in the prediction of atherosclerotic disease. New perspectives based on the Framingham study. Ann Intern Med.

[18] Holmes MV, Asselbergs FW, Palmer TM (2015). Mendelian randomization of blood lipids for coronary heart disease. Eur Heart J.

[19] Baigent C, Blackwell L, Emberson J (2010). Efficacy and safety of more intensive lowering of LDL cholesterol: a meta-analysis of data from 170,000 participants in 26 randomised trials. Lancet.

[20] Cannon CP, Blazing MA, Giugliano RP (2015). Ezetimibe added to statin therapy after acute coronary syndromes. N Engl J Med.

[21] Sabatine MS, Giugliano RP, Keech AC (2017). Evolocumab and clinical outcomes in patients with cardiovascular disease. N Engl J Med.

[22] Alkhalil M, Chai JT, Choudhury RP (2017). Plaque imaging to refine indications for emerging lipid-lowering drugs. Eur Heart J Cardiovasc Pharmacother.

[23] Ross R, Glomset JA (1976). The pathogenesis of atherosclerosis (first of two parts). N Engl J Med.

[24] Ross R, Glomset JA (1976). The pathogenesis of atherosclerosis (second of two parts). N Engl J Med.

[25] Taylor KE, Glagov S, Zarins CK (1989). Preservation and structural adaptation of endothelium over experimental foam cell lesions. Quantitative ultrastructural study. Arteriosclerosis.

[26] Davignon J, Ganz P (2004). Role of endothelial dysfunction in atherosclerosis. Circulation.

[27] Nielsen LB, Nordestgaard BG, Stender S, Kjeldsen K (1992). Aortic permeability to LDL as a predictor of aortic cholesterol accumulation in cholesterol-fed rabbits. Arterioscler Thromb.

[28] Schwenke DC, St Clair RW (1993). Influx, efflux, and accumulation of LDL in normal arterial areas and atherosclerotic lesions of white Carneau pigeons with naturally occurring and cholesterol-aggravated aortic atherosclerosis. Arterioscler Thromb.

[29] Carew TE, Pittman RC, Marchand ER, Steinberg D (1984). Measurement in vivo of irreversible degradation of low density lipoprotein in the rabbit aorta. Predominance of intimal degradation. Arteriosclerosis.

[30] Fisher EA, Feig JE, Hewing B, Hazen SL, Smith JD (2012). High-density lipoprotein function, dysfunction, and reverse cholesterol transport. Arterioscler Thromb Vasc Biol.

[31] Zanoni P, Khetarpal SA, Larach DB (2016). Rare variant in scavenger receptor BI raises HDL cholesterol and increases risk of coronary heart disease. Science.

[32] Lincoff AM, Nicholls SJ, Riesmeyer JS (2017). Evacetrapib and cardiovascular outcomes in high-risk vascular disease. N Engl J Med..

[33] Rohatgi A, Khera A, Berry JD (2014). HDL cholesterol efflux capacity and incident cardiovascular events. N Engl J Med.

[34] Ruparelia N, Chai JT, Fisher EA, Choudhury RP (2017). Inflammatory processes in cardiovascular disease: a route to targeted therapies. Nat Rev Cardiol.

[35] Moore KJ, Sheedy FJ, Fisher EA (2013). Macrophages in atherosclerosis: a dynamic balance. Nat Rev Immunol.

[36] Rong JX, Shapiro M, Trogan E, Fisher EA (2003). Transdifferentiation of mouse aortic smooth muscle cells to a macrophage-like state after cholesterol loading. Proc Natl Acad Sci U S A.

[37] Vengrenyuk Y, Nishi H, Long X (2015). Cholesterol loading reprograms the microRNA-143/145-myocardin axis to convert aortic smooth muscle cells to a dysfunctional macrophage-like phenotype. Arterioscler Thromb Vasc Biol.

[38] Allahverdian S, Chehroudi AC, McManus BM, Abraham T, Francis GA (2014). Contribution of intimal smooth muscle cells to cholesterol accumulation and macrophage-like cells in human atherosclerosis. Circulation.

[39] Hansson GK, Hermansson A (2011). The immune system in atherosclerosis. Nat Immunol.

[40] Akbar N, Digby JE, Cahill TJ, et al. Endothelium-derived extracellular vesicles promote splenic monocyte mobilization in myocardial infarction. JCI Insight 2017;2. doi: 10.1172/jci.insight.93344. 10.1172/jci.insight.93344 PMC5621885 28878126

[41] Rautou PE, Leroyer AS, Ramkhelawon B (2011). Microparticles from human atherosclerotic plaques promote endothelial ICAM-1-dependent monocyte adhesion and transendothelial migration. Circ Res.

[42] Xu J, Lu X, Shi GP (2015). Vasa vasorum in atherosclerosis and clinical significance. Int J Mol Sci.

[43] Fitzgibbons TP, Czech MP (2014). Epicardial and perivascular adipose tissues and their influence on cardiovascular disease: basic mechanisms and clinical associations. J Am Heart Assoc.

[44] Piepoli MF, Hoes AW, Agewall S (2016). European Guidelines on cardiovascular disease prevention in clinical practice: The Sixth Joint Task Force of the European Society of Cardiology and Other Societies on Cardiovascular Disease Prevention in Clinical Practice (constituted by representatives of 10 societies and by invited experts): Developed with the special contribution of the European Association for Cardiovascular Prevention & Rehabilitation (EACPR). Eur Heart J.

[45] No authors listed. Randomised trial of cholesterol lowering in 4444 patients with coronary heart disease the Scandinavian Simvastatin Survival Study (4S). Lancet. 1994;344:1383–9. 7968073

[46] Cannon CP, Braunwald E, McCabe CH (2004). Intensive versus moderate lipid lowering with statins after acute coronary syndromes. N Engl J Med.

[47] Ridker PM, Danielson E, Fonseca FA (2008). Rosuvastatin to prevent vascular events in men and women with elevated C-reactive protein. N Engl J Med.

[48] LaRosa JC, Grundy SM, Waters DD (2005). Intensive lipid lowering with atorvastatin in patients with stable coronary disease. N Engl J Med.

[49] Ridker PM, Danielson E, Fonseca FA (2009). Reduction in C-reactive protein and LDL cholesterol and cardiovascular event rates after initiation of rosuvastatin: a prospective study of the JUPITER trial. Lancet.

[50] Davignon J (2004). Beneficial cardiovascular pleiotropic effects of statins. Circulation.

[51] Jarcho JA, Keaney JF (2015). Proof that lower is better--LDL cholesterol and IMPROVE-IT. N Engl J Med.

[52] Lambert G, Sjouke B, Choque B, Kastelein JJ, Hovingh GK (2012). The PCSK9 decade. J Lipid Res.

[53] Giugliano RP, Pedersen TR, Park JG (2017). Clinical efficacy and safety of achieving very low LDL-cholesterol concentrations with the PCSK9 inhibitor evolocumab: a prespecified secondary analysis of the FOURIER trial. Lancet.

[54] Evaluation of cardiovascular outcomes after an acute coronary syndrome during treatment with alirocumab (ODYSSEY OUTCOMES). American College of Cardiology Congress; Orlando, FL, USA; Mar 10-12, 2018

[55] Ridker PM, Everett BM, Thuren T (2017). Antiinflammatory therapy with canakinumab for atherosclerotic disease. N Engl J Med.

[56] Mohler ER, Ballantyne CM, Davidson MH (2008). The effect of darapladib on plasma lipoprotein-associated phospholipase A2 activity and cardiovascular biomarkers in patients with stable coronary heart disease or coronary heart disease risk equivalent: the results of a multicenter, randomized, double-blind, placebo-controlled study. J Am Coll Cardiol.

[57] Serruys PW, Garcia-Garcia HM, Buszman P (2008). Effects of the direct lipoprotein-associated phospholipase A(2) inhibitor darapladib on human coronary atherosclerotic plaque. Circulation.

[58] Alkhalil M, Choudhury RP (2017). Evolocumab added to statins to reduce progression of coronary atherosclerosis. JAMA.

[59] Lipinski MJ, Benedetto U, Escarcega RO (2016). The impact of proprotein convertase subtilisin-kexin type 9 serine protease inhibitors on lipid levels and outcomes in patients with primary hypercholesterolaemia: a network meta-analysis. Eur Heart J.

